# Comparative Analysis of Waste Materials for Their Potential Utilization in Green Concrete Applications

**DOI:** 10.3390/ma15124180

**Published:** 2022-06-13

**Authors:** Kaushal Kumar, Saurav Dixit, Rishabh Arora, Nikolai Ivanovich Vatin, Jarnail Singh, Olga V. Soloveva, Svetlana B. Ilyashenko, Vinod John, Dharam Buddhi

**Affiliations:** 1 School of Engineering and Technology, K.R. Mangalam University, Gurugram 122103, India; ghanghaskaushal@gmail.com (K.K.); jarnailnith@gmail.com (J.S.); 2 Peter the Great St. Petersburg Polytechnic University, 195251 Saint Petersburg, Russia; vatin@mail.ru; 3 Lovely Professional University, Punjab 144411, India; 4 Institute of Heat Power Engineering, Kazan State Power Engineering University, 420066 Kazan, Russia; solovyeva.ov@kgeu.ru; 5 Basic Department of Trade Policy, Plekhanov Russian University of Economics, 117997 Moscow, Russia; svetavol@yandex.ru; 6 Amity University Noida, Noida 201301, India; vvinodjohn@yahoo.com; 7 Uttaranchal University, Uttarakhand 248007, India; dbuddhi@gmail.com

**Keywords:** waste materials, green concrete, strength

## Abstract

The utilization of solid waste in useful product is becoming a great deal of worth for individuals, organizations, and countries themselves. The powder of waste glass and silica fumes are also considered major waste materials across the globe. In this paper, the physico-chemical, thermal, and morphological properties of both waste powders are investigated in order to determine their suitability for use as a partial replacement for cement in basic concrete. They are suitable for use in concrete due to their pozzolanic and other basic properties. Extensive testing, in terms of the compressive strength test, the slump test, and the flexural strength test, has been carried out to study the replacement of cement in the range of 5–15% by waste glass powder for curing ages of 7 and 28 days. The FTIR analyses of both materials are studied for determining the effect of characteristics of chemical bonding and intense bands with bending vibrations of O–Si–O bonds. Experimental results indicate towards the potential utilization of wastes in concrete in terms of green concrete.

## 1. Introduction

**Figure 1:**
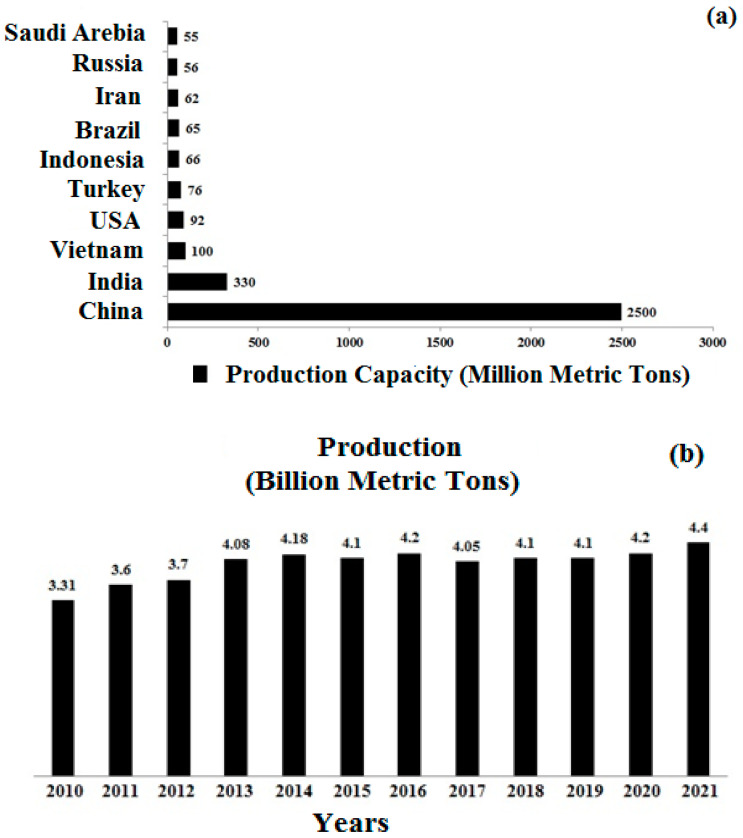
(**a**) Worldwide production scenario of cement and (**b**) production of cement during last decade [3].

The principal environmental challenges created by the emission of carbon dioxide (CO_2_) are global warming and climate change [1]. During the production of cement, a large amount of CO_2_ is emitted, which makes the construction industry the leading industry in causing the most CO_2_ emissions [2]. In 2016, it was reported that the cement industry emitted 522 million tonnes of CO_2_. [3]. The manufacturing of one tonne of cement is projected to release around 0.8 tonnes of CO_2_ into the atmosphere. Cement production is expanding at a steady rate of 2.5 percent per year over the world [4]. Figure 1 [3] shows that India is the world’s second-largest cement manufacturer after Europe.

Researchers throughout the world are looking for novel materials that can replace cement completely or partially [5]. Since the previous decade, many supplemental cementitious materials have been studied as cement replacements [6]. One billion tonnes of fly ash and 360 billion tonnes of ground-granulated blast-furnace slag are made each year in the United States, but only a small amount can meet the growing demand. As a result, finding other materials to replace cement has become a global necessity [7,8,9,10]. In order to check the feasibility, solid waste materials are also being explored in this research for the purpose of replacing cement and evaluating their potential for use in construction applications.

### 1.1. Waste Glass Powder

Due to having non-decomposition material, the generation of non-degradable waste is causing problems because it requires costly heat treatments, chemical treatments, environmental issues, and a huge area during disposal. These wastes include metallic, plastic, fiber, and glass residual, etc. [11]. A scrap of metallic and plastics undergoes the recycling process but the waste glass causes concerning issues.

Currently, material glass is used in the electronic sector to create a variety of screen-based goods such as LCDs, TVs, and tablets. Mirrors, hinged objects, and other similar items are used in the civil/construction businesses. In addition, they have uses in the food and medical industries, ranging from surgical equipment to beverage bottles [10]. As a result, the glass sector has a variety of possible examples around the world. Every product has a specific use period after which it is considered garbage. However, a considerable number of worthless objects, as well as defective or broken packaging glass, is transported to landfills, essentially freeing up space or land for more pressing needs [12]. As a result, the consumption countries must develop a better policy for reusing these trash glasses. According to a report, global waste glass powder production is around 130 Mt, with the EU (European Union) and China producing 65 Mt and the United States producing 20 Mt [13,14,15]. Its massive production has negative environmental consequences, and its disposal in landfills has become expensive. Because of its dependability, adaptability, and durability, glass powder is widely utilised in a variety of products around the world. Glass products are mostly made from recycled materials, and they may be recycled multiple times without affecting their chemical phases or arrangement [16]. Glass recycling begins with the melting of a combination of soda ash, silica, calcium carbonate (CaCO_3_), and recycled glass fragments. When glass powder reacts with calcium hydroxide, it exhibits a pozzolanic behaviour, which is enhanced by a higher surface area for the reactions [17]. According to studies, glass has a better chemical behaviour than other common materials [18,19]. Furthermore, it was discovered that fine milling glass powder increases the interactions between glass and cement hydrates, and that it can be used as a cement additive [20]. There have also been numerous pieces of research on the pozzolanic activity of tiny glass powder particles in concrete [21,22]. Glass powder generally exhibits a pozzolanic reaction at a slower rate than cement hydration, depending on its fineness [23]. The extensive literature shows that glass powder has potential for utilization in concrete applications, so it is necessary to conduct an extensive study for more utilizations of waste glass powder as the replacement of cement.

### 1.2. Silica Fume

**Figure 2:**
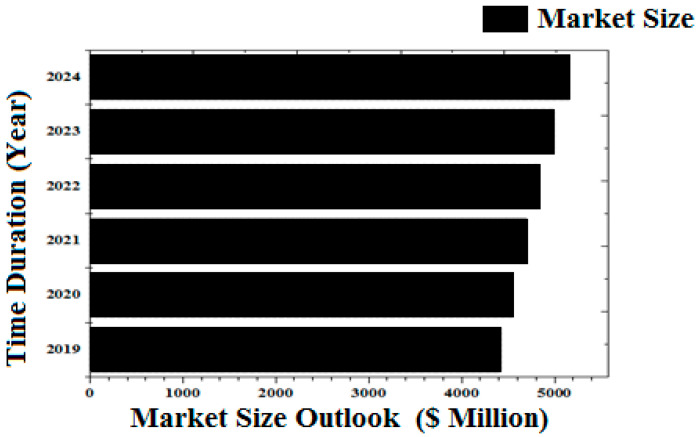
Forecasting as well as present and past market size scenario [37].

Silica fume is a by-product of the manufacturing of silicon metal or ferrosilicon alloys. The oxidized silicon vapour from the furnace condenses into small spherical particles, which are collected in silos as a dry powder [24]. Dry powder is frequently made up of agglomerates that can be several millimetres in size and are difficult to disperse into individual silica fume spheres using conventional concrete mixing methods [25]. Silica fume has a lot of surface area, approximately 20,000 m^2^/kg. The surface area of portland cement determined by nitrogen absorption, for example, is roughly 1500 m^2^/kg. The amorphous character of SiO_2_ in silica fume, which gives great potential to react in Pozzolanic reactions, is a particularly favourable attribute when combined with the high surface area values [26]. The surface area is crucial, and it can be improved by de-agglomerating the raw micro silica powder precisely [26,27]. According to Committee 234 of the American Concrete Institute (ACI) [28], condensed silica fume is produced by blowing compressed air from underneath and scattering fume particles in a silo, causing them to agglomerate. The popularity of condensed silica fume has risen due to the belief that agglomerates are brittle and will easily dissolve and shatter when mixed with aggregates. Further investigations, according to the ACI Committee 116R [29], have revealed that this occurs infrequently, and that major fracturing of the agglomerates does not disintegrate completely in hardened concrete. According to studies, silica fume agglomerates are larger than cement particles in concrete, preventing the performance advantages projected from pore-size refinement and matrix densification. Hooton’s initial findings [30] suggested that silica fume may be used to control the alkali–silica reaction (ASR), prompting a rise in the use of silica fume in a range of applications. However, investigations have shown that the existence of agglomerates in condensed silica fumes, which do not dissolve or scatter uniformly during concrete mixing, can impair the previously claimed ASR-related advantages [31,32,33,34,35,36]. Numerous researchers reported that the production of silica fume is increasing day by day. In addition, the market size was also forecasted, as shown in Figure 2.

## 2. Methodology

Two distinct waste materials with the same quantity have been used for testing and comparative analysis of the concrete, e.g., glass milling (WGP) and silica fume. Physical properties of waste materials sample are listed in Table 1.

The comparison is based on the use of waste resources to replace a tiny portion of portland cement. This replacement must not modify the water content of the concrete, as it does with ordinary concrete. Water, ordinary and portland cement, aggregates, and waste material are the key components of this concrete mixture. During this process, suitable cooling processes are used together with material testing to achieve the desired strength. After selecting the optimal w/c ratio through suitable mix trials, the mix design is created. The proportions of the materials are likewise appropriate to the design.

### Sample Preparation and Experimentation

The M-20 mix percentage is used to prepare samples. In M-20, M denotes Mix and 20 refers to the characteristic strength (f_ck_) of that mix, i.e., 20 MPa, cement, sand, and aggregates are used for mixing in the ratio of 1:1.5:3. M-20 signifies mixture of cement, sand, and aggregate that are prepared in such a manner that a cement concrete cube of size 15 cm × 15 cm × 15 cm is formed with characteristic strength (f_ck_) of 20 MPa while examining it after being cured for 28 days. The characteristic strength (f_ck_) signifies the strength under which not over 5% of test results are predictable to fail.

Table 2 lists the rest of the information. 24 cubes of each waste material were constructed to investigate the impact of WGP and silica fume as partial cement replacements. For both waste products, the water to cement and cement to sand ratios are 0.6 and 1:3, respectively, throughout the design process.

The combination was produced with typical or standard proportions for testing purposes (M-20). The initial sample was made or the first mix was made entirely of cement, with no waste material substituted. Different waste materials are used to make different specimens, but the requirements and standards remain the same. In other words, the standards for preparing waste glass powder samples are comparable to those for silica fume samples. Cement is partially replaced in concrete by waste materials at a 5 percent ratio. The WMx-1 mix represents a 5% substitution of waste material for cement. As a result, Mx-0 contains 100% cement, whereas Mx-1 includes 95 percent cement and 5% waste material. Mx-2 includes 90% cement and 10% waste material, whereas Mx-3 contains 85% cement and 15% waste material. Samples of waste material cubes measuring 150 mm × 150 mm × 150 mm were made (mm). The whole design of the mixing proportion of samples is provided in Table 3, with WGP samples identified by the letter “W” and silica fume samples marked by the letter “S”.

Six samples (three for each set and each material) for each test have been prepared with replacement of cement from waste material and without replacement of any cement particle, respectively.

Firstly, concrete paste has been prepared with a proper proportion of the materials. To analyze the properties of prepared concrete the specimens were cast and cured prior. For the curing stage of the sample, proper environmental conditions have been maintained till the completion of the experiments. A humidity chamber is used for keeping the samples for one day from the setting, where the temperature range is maintained as 20–21 °C and humidity is about 96–97%. Ideal standard testing conditions (T = 20 °C and Relative humidity = 100%, with water immerged sample) were also used for samples after the completion of the primary stage. The conditions were maintained until the testing duration (7 and 28 days).

**Figure 3:**
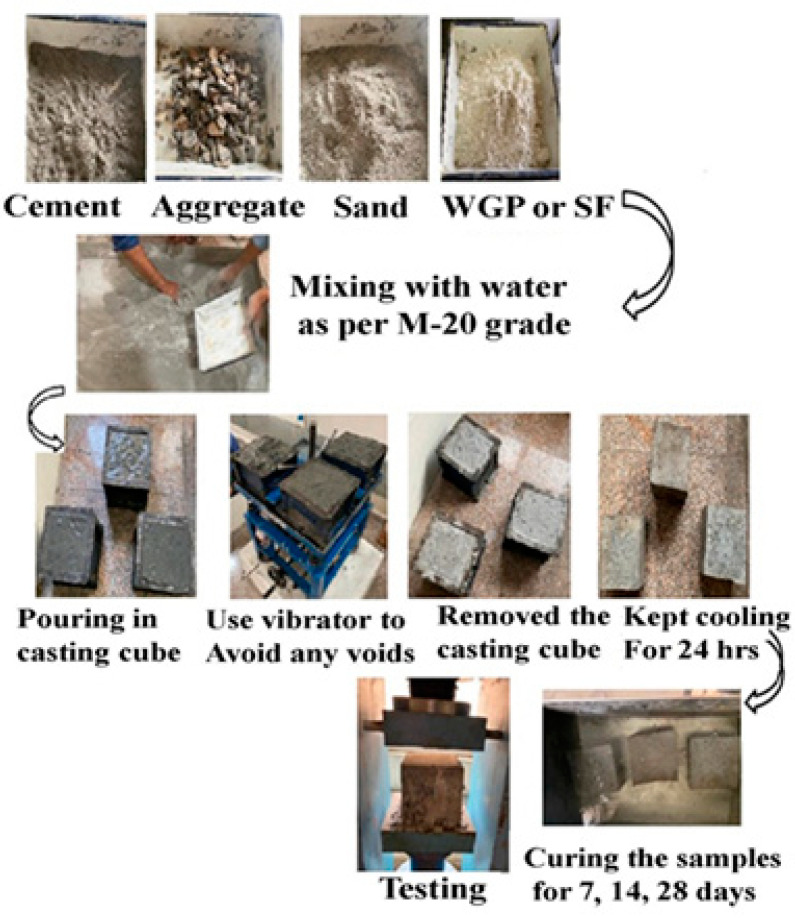
Process of formation of green concrete through the waste.

The complete procedure of testing has been shown in Figure 3. The procedure involves the collection of constituents, and the mixing, pouring, curing, and testing process to complete the procedure. The prepared sample was put to the test for two different types of concrete tests: fresh concrete and secondly hardened concrete. The slump test and the compaction factor test are two primary tests. Secondary testing, on the other hand, includes the compressive strength test (Make: Aimil *CTM* IS: 14858 (2000) with an accuracy of ±1%) and the flexural strength test (Make: Aimil FTM, IS 516 BS1881, with an accuracy of ±1%). For strength testing, a compression testing machine with a capacity of 2000 KN was used. The ASTM standards were used to conduct the slump test while compaction factor has been determined based on IS1199:1999 standards. Similarly, compression strength test was performed with standards ASTME-9 while flexural strength test was performed with standards ASTMC-293. The average of three tests has been calculated for the average compression strength and average flexural strength.

## 3. Results and Discussion

**Figure 4:**
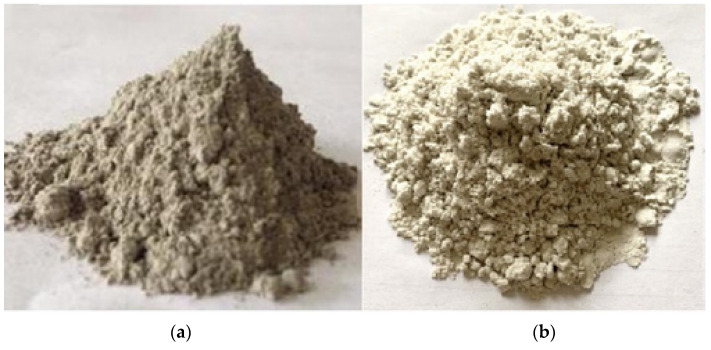
Real pictorial view of (**a**) Silica Fume and (**b**) Waste Glass Powder.

To check the feasibility in construction and other applications, both waste materials have undergone extensive characterizations. The sample of SF was taken from the local site of the Hisar (Haryana) region of India. The sample was cleaned properly and put into the oven at 150 °C for removal of the moisture content prior from being used in the different laboratory level tests. It looks like grey powder to the naked eye, as seen in Figure 4a. In the instance of waste glass powder, however, white transparent bottles were collected and properly cleansed with clean water. With the use of a roller ball mill, clean bottles were crushed and finely ground into powder for up to 1 h, and the powder form of waste glass was stored in a separate bucket. Figure 4b depicts the true image of WGP, which includes a crushed bottle and a powdered form of glass. The WGP appears to be a pure white powder, according to the researchers. In the basic concrete, this WGP and silica fume are utilised to substitute cement.

**Figure 5:**
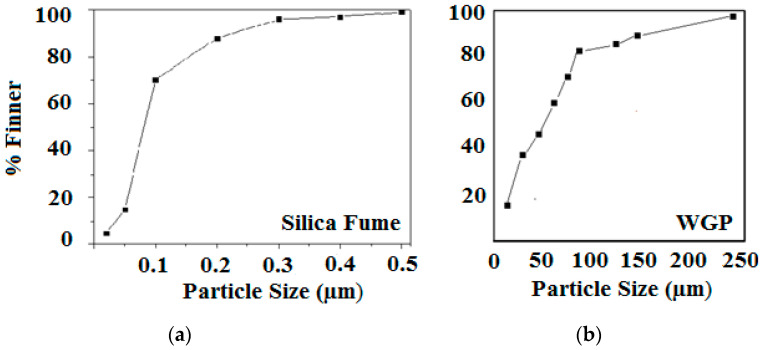
Particle Size Distribution of (**a**) Silica Fume and (**b**) Waste Glass Powder.

To measure the fineness or particles size of the materials, sieve analysis has been done. The particle size distribution of both waste items is determined using a mechanical sieve shaker. Standard sieves of brass with a metallic net of different grades are used. The sieves are arranged in such a way that the pan collects finer particles. Figure 5a,b illustrate the detailed particle size distributions of silica fume and WGP, respectively. It was discovered that over 70% of silica fume particles were finer than 0.10 microns and approximately 96% were finer than 0.30 microns. While around 48 percent of WGP particles are finer than 45 microns and approximately 40% are finer than 32 microns. For testing reasons, the finest form of particles (0.1 µm) were deposited separately and used to substitute cement in the concrete.

**Figure 6:**
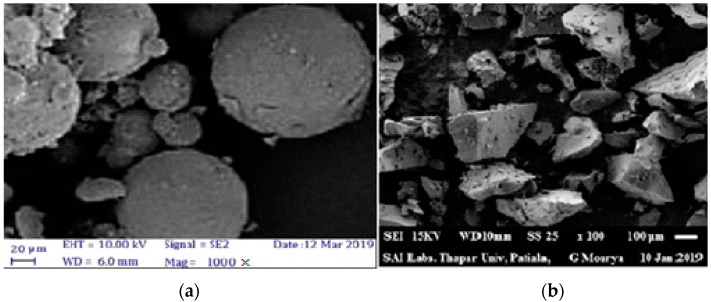
Scanning Electron Microscopy (**a**) Silica Fume and (**b**) Waste Glass Powder.

The morphology of these waste items is also studied using the finest particles or the particles collected through PAN by using the ASTM D422 standard. The morphology and elemental content of the materials were studied using a scanning electron microscopy-energy dispersive X-ray spectroscope (Model: JEOL, 6510 LV). Figure 6a,b show SEM images of silica fume and WGP, respectively.

Both materials’ particles have a smooth surface morphology, according to the findings. In comparison to smooth spherical, agglomerated particles of silica fume, WGP particles are shredded, uneven, and angular in shape.

**Figure 7:**
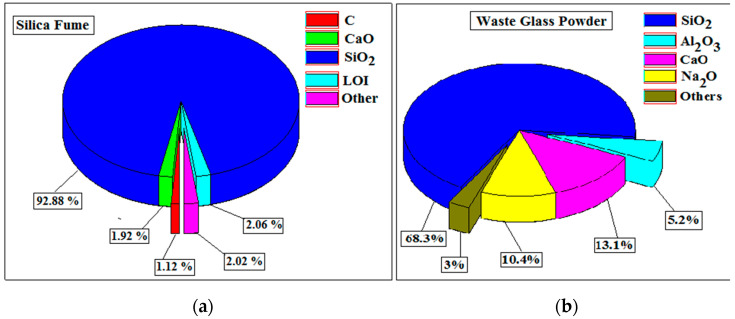
Chemical composition of (**a**) Silica fume and (**b**) Waste Glass Powder.

The elemental composition of materials is determined with an energy-dispersive X-ray spectroscope (EDS). Figure 7a,b show the exact chemical compositions of silica fume and WGP. The silica fume powder was found to be predominantly enriched with silica (SiO_2_), with a tiny fraction of CaO and C having a marginal 2.06% LOI. CaO has 1.92 percent and C has 1.12 percent, while Na_2_O, MgO, K_2_O, Al_2_O_3_, and other oxides have 2.02 percent.

In the instance of WGP, it was revealed that the powder is primarily enriched with silica and calcium, with modest amounts of Na and Al. Both materials were found to be mostly enriched in amorphous silica (SiO_2_), which has a high specific surface area. They can react with portlandite and exhibit pozzolanic properties under these conditions [14,15,18,25]. This could lead to the use of both elements in concrete in place of expensive cement.

**Figure 8:**
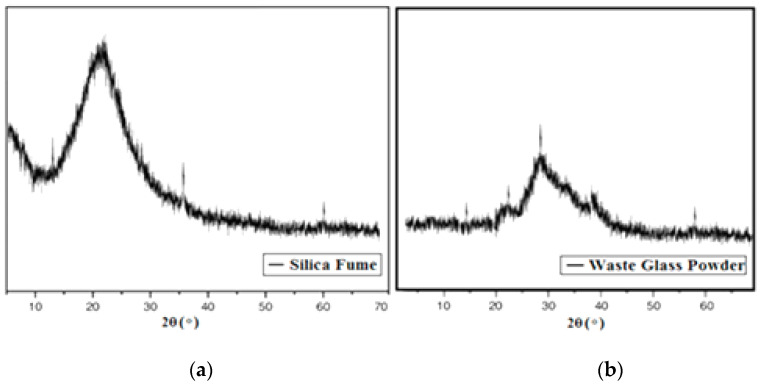
XRD of (**a**) Silica fume and (**b**) Waste glass powder.

XRD characterization of the powders was performed with the specifications utilising monochromatic Cu K-radiation operating at 50 kV and 80 mA to explore the availability of phases in the materials. For the testing, a Philips X-ray diffraction with an angular speed of 2°/min and a step of 0.02° was used. During the testing, the temperature range for two was 4°–60°. In Figure 8, an XRD pattern of silica fume is depicted. (**a**) A single intense peak is observed around 21.6°, which confirmed the presence of silica fumes [38]. The presence of a broad peak demonstrated that the silica fumes used in this research work are amorphous because silica fumes are composed of silica, which is non-crystalline [25]. Therefore, a broad peak is reflected in the XRD pattern. The XRD pattern of waste glass powder, on the other hand, is likewise shown in Figure 7b. The amorphous nature, which is characteristic of glassy materials, is also blamed for the large, diffracted peaks. The WGP does not contain any high peaks; rather, a widespread diffraction peak between 20° and 40° was found, indicating the presence of a glassy phase. The highest peak of SiO_2_ was found at 27°, while the maximum peak of CaO was found between 23° and 35°. A previous study has provided similar results [17].

**Figure 9:**
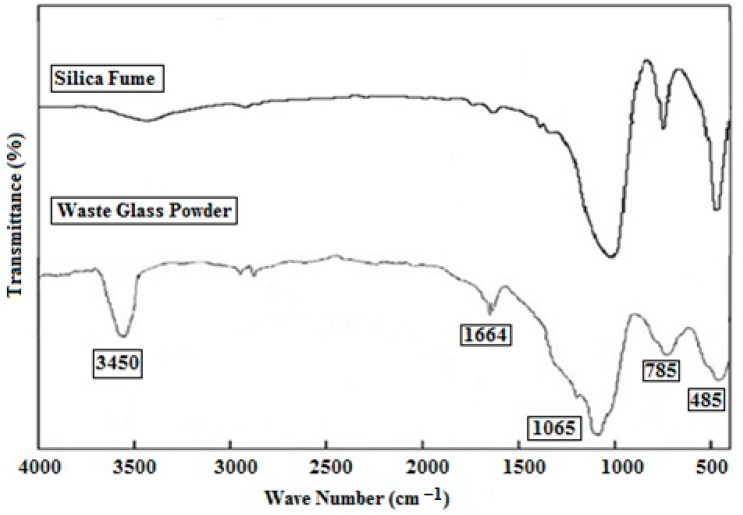
FTIR analysis of silica fume and waste glass powder.

FTIR transmission spectrum of silica fume and WGP are shown in Figure 9. This measurement was carried out to demonstrate the characteristics of chemical bonding. As discussed in the XRD analysis that silica is the major content of silica fumes, hence, characteristic peaks of silica are observed in the FTIR spectrum. The Si–O–Si asymmetric stretching bonding is responsible for the exceptionally strong absorption band detected around 1085 cm^−1^. This band is not only very bright, but it also has asymmetric bands that can be used as a diagnostic sign for the presence of silica in a sample. The band seen at 486 cm^−1^ is linked to the bending vibration of the O–Si–O bond. Furthermore, the 719 cm^−1^ band is caused by in-plane bending vibrations of Si–O bonds [25]. The FTIR spectra of glass waste revealed a broad and intense band at 1085 cm^−1^, which corresponds to the Si–O asymmetric stretching vibrations in the SiO_4_ tetrahedral groups, and another at 485 cm^−1^, which corresponds to the O–Si–O bending vibrations in the SiO_4_ groups. A broad band was detected at 1646 and 3450 cm^−1^, respectively, ascribed to the δ–H–O–H and reticular v-OH functional groups.

One of the most important features of concrete mix design is workability, which is the time and work it takes to handle, install, compact, and finish new concrete. Slump, mini-slump flow, and slump flow tests are the most common procedures for testing the workability of concrete. The most essential factor in determining workability is the amount of water present. Slump tests were performed on both samples in this investigation. The slump test and compaction factor test were performed independently for each mix design of the concrete sample before the other tests.

**Figure 10:**
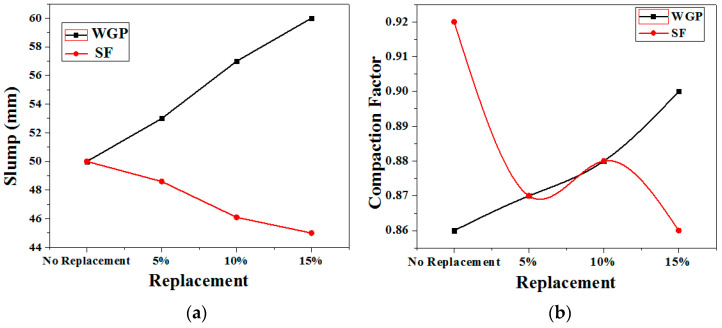
(**a**) Slump Test and (**b**) Compaction Factor test for both samples.

Figure 10 depicts the impact of replacement on workability, slump flow, and compaction factor. In the case of silica fume, it has been observed that the slump decreases as the percentage of silica fume replaced in the concrete increases. In concrete, it has been noticed that as the percentage replacements of silica fume increase from 0% to 15%, the slump flow decreases from 50 mm to 45 mm. With an increase in percentage substitutions of silica fume in concrete, the result indicates a declining trend in workability. With a rise in percentage substitutions of silica fume in concrete, the slump value, as well as the compression factor, lowers. Because of its large surface area, silica fume with a high percentage reduces workability [39]. This could be due to the small particle size, which allows a lot of water to absorb on the surface [39].

Furthermore, silica fume’s spherical form reduces water absorption; antiparticle friction is reduced since the particles serve as little bearings. The water-reducing ingredient is widely used with silica fume in order for it to compact easily with cement grains and act as a lubricant [40,41]. However, compared to silica fume, the replacement of WGP has a negative effect on workability. It appears that workability improves as the ratio of WGP replacement in concrete increases. The slump value, as well as the compaction factor, improves as the percentage of WGP in the concrete is increased. The slump flow in concrete increases from 50 mm to 60 mm as the percentage replacement of WGP increases from 0 to 15%. With an increase in the substitution of WGP in basic concrete, a rising trend in workability was noticed.

According to the findings, the slump flow of concrete containing glass powder as a replacement for cement increases as the glass powder content increases. The glassy surface and low water absorption behaviour of glass powder could cause an increase in slump. Furthermore, glass powder has coarse particles when compared to cement, which may have led to the increase in a slump. Glass powder has a lower water absorption rate and a smoother texture than cement particles, which increases workability [42,43,44]. Another explanation for the increase in workability is the dilution of the cement. Because of the aforementioned reasons, cement hydration products grow more slowly in the early minutes. As a result, there are not enough products on the market that combine various particles. The overall surface area of the cement and glass powder mixture is reduced because glass powder has a lower specific surface area than cement [45,46,47,48,49].

As a result of the reduced water demand for particle surface lubrication, the slump flow increases. On the other hand, the value of the compaction factor is found as 0.86 to 0.9 for WGP and 0.92 to 0.86 for silica fume concerning the replacement of 0 to 15% waste from basic concrete. The compaction factor of concrete decreases with an increase in percentage replacements of silica fume in it, while in the case of WGP it shows a different or opposite trend as shown in Figure 10b.

Following initial testing, each sample was evaluated for compressive and flexural strengths. When compared to other samples, the WMx-3 sample is expected to have a higher or maximum Compress (WMx-0, 1, and 2). Similarly, when compared to other samples, the SMx-3 sample has a greater or maximum compressive strength (SMx-0, 1, and 2). The results show that when 15% of the cement in both samples is replaced, the compressive strength of the concrete increases.

**Figure 11:**
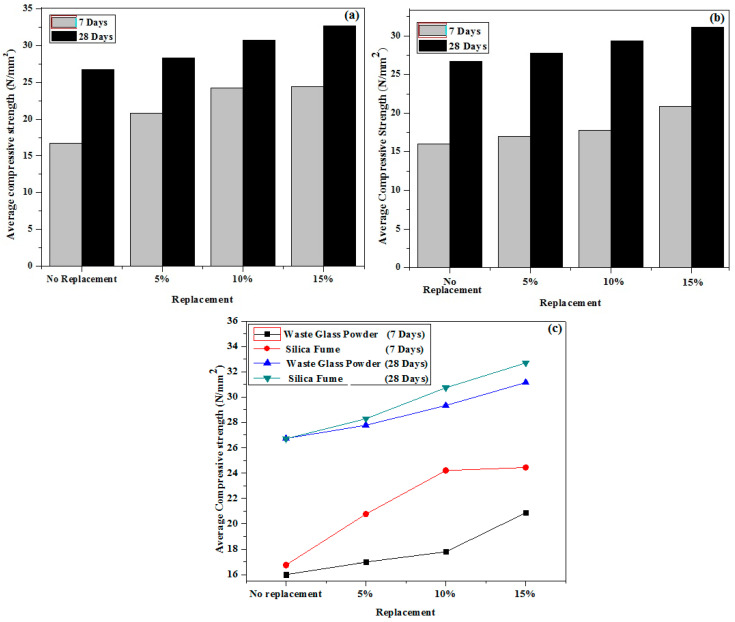
(**a**) Silica fume, (**b**) WGP, and (**c**) comparison of both waste products variation in average compressive strength of different samples and durations.

When compared to any of the WMx samples, the maximum compressive strength was found in samples SMx-3 (24.44 N/mm^2^) for 7 days and SMx-3 (32.69 N/mm^2^) for 28 days. It was discovered that when adding 15% WGP, the average compressive strength improves by approximately 31% in 7 days and 16.5 percent in 28 days, while adding 15% silica fume, the average compressive strength increases by approximately 46% in 7 days and 16% in 28 days, respectively. Mx-0 < Mx-1 < Mx-2 < Mx-3. Figure 11 depicts the variation in the average compressive strength of different samples and periods. The results showed that both waste products may be used to replace cement to a maximum of 15%. When compared to WGP, however, a sample of silica fume had the highest compressive strength. This could be attributed to the pozzolanic characteristics of silica fume [50,51,52,53,54,55,56].

**Figure 12:**
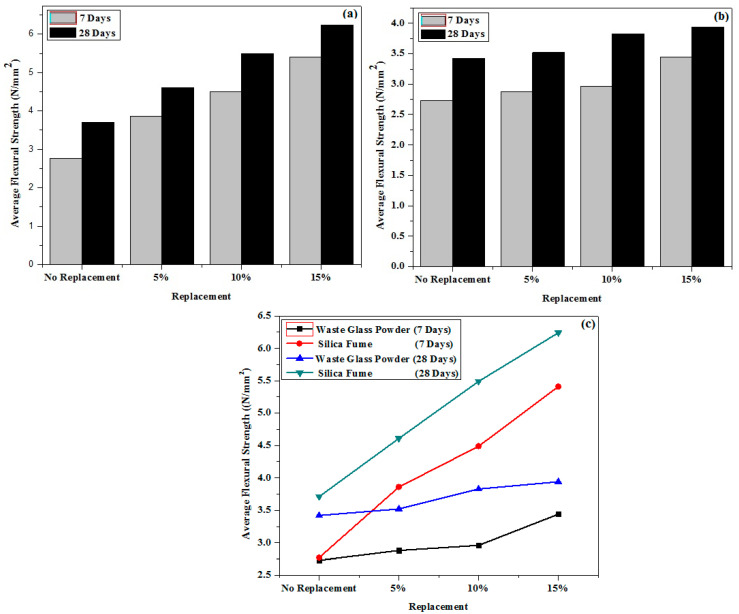
(**a**) Silica fume, (**b**) WGP, and (**c**) comparison of both waste products variation in average flexural strength of different samples and durations.

For the flexural strength test, a similar set of trials was carried out. The average flexural strength of different samples and durations is shown in Figure 12. The flexural strength of concrete containing 15% waste material is higher than that of the reference concrete, as can be shown. In comparison to the no-replacement case, the highest increase in AFS was found when 15% of the cement was replaced with waste materials (WGP and silica fume). The increase in AFS of the WGP (WMx-3) sample was determined to be approximately 26 percent at the age of 7 days, and about 15.2 percent at the age of 28 days. Similarly, the increase in AFS of the silica fume (WMx-3) sample was determined to be around 95 percent at the age of 7 days, and approximately 68 percent at the age of 28 days. For both waste sources, the order of increasing AFS in the concrete sample is as follows: Mx-0 < Mx-1 < Mx-2 < Mx-3. The maximum replacement (15%) of cement from WGP exhibits the greatest improvement in AFS, according to the findings. It was also discovered that the improvement in the AFS for silica fume samples is substantially more than for the tougher WGP sample. The findings show comparable patterns to those seen in the research [8,13].

According to the findings, the value of both strengths (CS and FS) is lower in the curing age of 7 days compared to the curing age of 28 days for both waste materials. During the investigation, however, the variation in increments was also noticed. The rate of improvement in the concrete’s compressive and flexural strength was substantially faster in the 7-day instance than in the 28-day example. The strength (CS and FS) of concrete improves with the curing age or time for any single design group, but at a slower rate than at the beginning. This could be owing to the waste materials’ delayed pozzolanic reactivity [10,16].

## 4. Conclusions

In this present work, the compressive strength of concrete was found to be increased with the incorporation of WGP and silica fume. In addition, the compressive strength and hardness of concrete were also affected by altering the waste material weight ratio. It was observed that after replacing 15% of the WGP, the average compressive strength of concrete rose by 31% after 7 days and by 16.5% after 28 days. In the case of 7 days, approximately 46% of the average compressive strength increases with the addition of 15% silica fume, while the improvement is 16% in the case of 28 days. When 15% of the concrete was replaced with silica fume, the improvement in concrete strength was substantially higher than when the same quantity of concrete was replaced with WGP. The slump value and compaction factor of both waste materials show opposite trends; in silica fume the value of the slump and compaction factor decreases with the replacements, which shows less workability, while in case of WGP, both values increase and are responsible for the good workability. Both the waste materials show better chemical bonding and pozzolanic properties with the replacements, that further tends to make them suitable for better alternative of cement in concrete. Hence, it was concluded that concrete strength increased with the replacement of cement content from WGP and silica fume.

## Figures and Tables

**Table 1:** Physical properties of waste materials sample.

Sr. No.	Physical Properties	Materials and Measure		Unit
		Silica Fume	Waste-Glass Powder	
1	Particle Size Distribution	<1	10–250	µm
2	Specific Gravity	2.22	2.94	-
3	Bulk Density	380	2540	kg/m^3^
4	Color	Grey	White	-
5	Surface area	16,000	3130	m^2^/kg

**Table 2:** Mix Design and Proportions with details.

Entity	Proportion
Cement (kg/m^3^)	399.2
Fine Aggregate (kg/m^3^)	672.8
Coarse Aggregate (kg/m^3^)	1097.2
Water (Litres/m^3^)	191.6
Water Cement Ratio	0.48
Mix Ratio	1:1.5:3
Proportion	M 20

**Table 3:** Concrete Mix design Summery.

Mix Designation	Cement	Glass Powder/Silica Fume	Fine Aggregate	Coarse Aggregate
Mx-0	100%	0%	100%	100%
WMx-1/SMx-1	95%	5%	100%	100%
WMx-2/SMx-2	90%	10%	100%	100%
WMx-3/SMx-3	85%	15%	100%	100%

## Data Availability

Not applicable.
